# Differential Subjective Experiences in Learners and Non-learners in Frontal Alpha Neurofeedback: Piloting a Mixed-Method Approach

**DOI:** 10.3389/fnhum.2018.00402

**Published:** 2018-10-23

**Authors:** Eddy J. Davelaar, Joe M. Barnby, Soma Almasi, Virginia Eatough

**Affiliations:** ^1^Department of Psychological Sciences, Birkbeck, University of London, London, United Kingdom; ^2^Centre for Neuroimaging Sciences, Institute of Psychiatry, Psychology, and Neuroscience, King’s College London, London, United Kingdom

**Keywords:** EEG neurofeedback, qualitative analysis, neurophenomenology, subjective experience, alpha oscillations

## Abstract

In a neurofeedback paradigm, trainees learn to willfully control their brain dynamics. How this is realized remains an open question. We evaluate the hypothesis that learning success is associated with a specific phenomenology. To address this proposal, we combined quantitative and qualitative analyses of a short neurofeedback training (NFT) session during which participants enhanced mid-frontal alpha power and were then subsequently interviewed about their experiences. We analyzed the electrophysiological data to determine learning success and classify trainees as learners and non-learners. The subjective experiences differed between the two groups and are best described along a trying-sensing continuum, with non-learners engaging effortfully with the task (e.g., “I will it [the bar] to move”) whereas learners reported more sensing of their inner (e.g., “Something inside my stomach”) and outer environment (e.g., “I was aware of the sound of the beeps”). In the process of piloting this mixed-method approach, we developed a classification system for the verbal reports. This system provides an explicit analytic framework which might guide future studies that aim to investigate the association between subjective experiences and NFT protocols.

## Introduction

In a neurofeedback paradigm, trainees learn to willfully control their brain dynamics. With the recent increase in commercially available tools for measuring brain and body functions to improve physical health and enhance mental performance, the field of applied neuroscience, and the domain of neurofeedback in particular, is enjoying a renewed interest from researchers of cognate disciplines. This interest brings to the neurofeedback research a wide range of neuroscience methodologies, such as measuring brain activation using functional magnetic resonance imaging (fMRI) (Sulzer et al., 2013; Emmert et al., 2016), functional near-infrared spectroscopy (fNIRS) (Sakatani et al., 2013; Marx et al., 2015), and magnetoencephalography (MEG) (Florin et al., 2014; Okazaki et al., 2015), to go alongside the traditional focus on electroencephalography (EEG). It also leads to revisiting research questions that earlier generations of researchers were unable to fully address due to the lack of research tools, methodologies, and paradigms that were yet to be developed. In this paper, we take up the long-standing question whether there exist differences in the subjective experience and strategies between trainees who are able and those who are unable to learn to change their EEG dynamics. To address this question, we combine quantitative and qualitative methodologies. Specifically, we present our mixed-method approach, which combines EEG profiling and thematic analyses of interviews, informed by phenomenological and hermeneutic principles, as a contribution to the reinvigorated neurofeedback research domain. Before presenting our study, we address the relevance of understanding subjective experiences and briefly review research that addressed the subject. We focus on the methods used in previous research and introduce the concept of neurophenomenology and the associated explicitation interview, which forms the basis of the work presented here.

### The Relevance of Understanding Subjective Experiences

Knowing the subjective experiences (i.e., the first-person sensations that reach conscious awareness) associated with certain EEG neurofeedback protocols that distinguish learners from non-learners can have considerable impact on NFT efficacy. In a recently proposed theory of neurofeedback learning, the subjective experience is a crucial factor contributing to neurofeedback success during follow up in the absence of explicit training using equipment (Davelaar, 2018). In particular, in the multi-stage theory of neurofeedback learning, the second stage involves the detection of a distinctive subjective representation that, as it is always paired with a reward signal, becomes a secondary reinforcer in the final stage. Thus, when the trainee attempts to enter the required EEG state and is not connected to the neurofeedback equipment, the subjective experience becomes the feedback signal. This feature, although not an obligatory one, is hypothesized to contribute to the long-term success of a neurofeedback intervention.

There have been several attempts to map the subjective experiences associated with NFT protocols, but no standardized methodology has been developed, making comparison across studies almost impossible. However, two recurrent approaches can be discerned. There is a tendency to ask trainees about their strategies, which are narrow subjective experiences centered on cognitive actions, and then count the number of participants that report a certain strategy (Nowlis and Kamiya, 1970; Hinterberger et al., 2005; Nan et al., 2012; Kober et al., 2013). The strategies in turn tend to be classified along an emotional valence axis. For example, Nan et al. (2012) trained individual alpha band frequency and asked trainees to write down the strategy used and its (perceived) effect after each of 20 training sessions. The strategies were divided into positive, neutral, and negative types. In contrast, Gruzelier (2014) measured mood as a consequence of training using post-training questionnaires. Although questionnaires do assess experiences, they provide the wording, leading to potential misinterpretations of labels and missed opportunities of unlabeled experiences.

Both approaches (asking about strategy use and post-training questionnaires) lack the necessary depth that a true qualitative methodology can provide. For example, in the study by Nan et al. (2012), it is unclear in what context “raining” (as part of the “nature” subtype) and “shopping” (as part of the “life” subtype) could both be considered “positive.” Nowlis and Kamiya (1970) instructed participants to find out on their own what strategy works for them to increase or decrease posterior alpha amplitude. They reported that (among others) *awareness*, *relaxation*, *not focusing*, and *letting go* were associated with increase in alpha. However, the verbal reports from the interviews were reduced to single-word labels. This means that *relaxation* could actually reflect “trying to relax” or “feeling relaxed,” which are subjectively different mental states. Kober et al. (2013) asked participants to write down their mental strategies after the first and last SMR or gamma training session. They found that those participants who reported no (specific) strategy at the end were shown to be successful learners. This might mean that either participants were “doing nothing” or were “doing a lot of different strategies.” In addition, the authors provided instructions to the participants (i.e., be mentally focused, while physically relaxed). Thus, having no specific strategy might even mean not adhering to the instructions given. To understand the meaning of participants’ responses, the context of the experiment needs to be taken into account.

The context in which the words are used is critical for the interpretative analysis. Edge and Lancaster (2004) attempted a hermeneutic analysis of the experience with NFT and its use in the context of performing music. They interviewed participants from the study by Egner and Gruzelier (2003) who were trained on a SMR/beta protocol (10 sessions) followed by an alpha/theta protocol (10 sessions). A single interview took place after all 20 training sessions were completed. Unfortunately, several methodological obstacles preclude giving too much weight to their findings in the context of addressing NFT success. First, the experiences reported were only those that were connected to music performance and not to NFT success. Second, the participants were not told about the impending interview, leading to difficulties in data acquisition. Third, the interval between the final training session and the interview could be weeks or even months. Yet, these caveats aside, Edge and Lancaster (2004) demonstrated that a qualitative approach that makes use of the context within which the verbal reports are generated can be integrated into a neurofeedback research program.

### Subjective Experience and Brain States

Understanding the differences in subjective experiences between learners and non-learners provides researchers with insight into the distribution of success rates. It also allows trainers to provide targeted instructions to trainees to speed up the learning and observe therapeutic benefits sooner. However, a number of challenges exist that make a simple “What did you do?” question ineffective. First, not all subjective experiences are readily verbalizable. Insofar that a subjective experience exists, a detailed phenomenological interview is needed to extract the experience lived by the trainee. Second, the ability to communicate subjective experience varies greatly across individuals. Extracting the information requires paying attention to the details of the interview procedure to support trainees in sharing their experiences. Third, not all trainees will have the exact same experience. As will be shown below, we developed a classification system based on responses from the interviews.

There is only scarce evidence showing associations between subjective *experiences* and EEG NFT success. Gruzelier et al. (2010) found that individuals tasked to increase SMR while keeping theta and beta low over Cz were able to indicate at what stage they recognized a difference in mental state, which was supported by the actual EEG analyses. This suggests an association between the EEG state and subjective experience in the context of NFT. However, the actual sensation experienced by the trainees on which the recognition was based was not investigated.

A relevant study outside the domain of EEG NFT was done by Garrison et al. (2013). They obtained verbal reports from ten highly experienced (18.4 ± 4.9 years) meditators after each of three meditation sessions and one neurofeedback session in the fMRI scanner. Whereas the first two sessions were meditation sessions, the third session required meditation plus noticing the association between the moment-to-moment subjective experience and the feedback display, which reflected the activation of the posterior cingulate cortex (PCC). The final session required direct manipulation of the display, specifically increasing PCC activation. Participants were asked how well the feedback corresponded with their experience and their strategy use to manipulate the feedback. They observed that PCC activation was associated with “distracted awareness” and “controlling,” whereas PCC deactivation was associated with “undistracted awareness” and “effortless doing.”

Associating subjective experiences with neural patterns falls in the realm of neurophenomenology. The working hypothesis of the neurophenomenological program inspired by the late Francisco Varela is that “phenomenological accounts of the structure of experience and their counterparts in cognitive science relate to each other through reciprocal restraints.” (Varela, 1996, p. 343). The basic premise is that experience is irreducible and that a disciplined understanding of it requires a second-person method which means the rigorous collection of first-person accounts of subjective experience through careful guiding and questioning from a researcher. Phenomenological methods provide a disciplined approach to achieving this. Bagdasaryan and Le Van Quyen (2013) view that neurofeedback is the perfect paradigm to investigate neurophenomenology, as it provides a window into the causal relations between brain states and subjective experiences.

### The Current Study

This study is part of the larger Birkbeck EEG Neurofeedback and Neurophenomenology (BENN) study (Davelaar et al., unpublished), which aims to develop an integrated mixed-method approach for neurofeedback research. The BENN study consisted of 10 sessions of neurofeedback training (NFT) on either mid-frontal theta or mid-central sensorimotor rhythm. The NFT sessions were preceded and succeeded by a session of cognitive testing. As part of the pre-NFT session, participants were introduced to the neurofeedback equipment and setup by experiencing a 5-min training block aimed to enhance frontal alpha power. After this short training block, participants were interviewed for approximately 10 min about their experiences during this training block. This study was carried out in accordance with the recommendations of the ethics board of Birkbeck, University of London, with written informed consent from each participant. All participants gave written informed consent in accordance with the Declaration of Helsinki. We report here on the analyses of these interviews. The main data from the BENN study addressed other research questions and will be presented elsewhere. Our aim in this paper is to develop a replicable analytical procedure that integrates subjective phenomenological experiences with objective EEG measurements. The utility of the approach is demonstrated by the unexpected, but understandable finding that differentiates learners from non-learners.

## Materials and Methods

Out of 25 participants, complete data were available from 17 participants. These were the participants who subsequently continued with the mid-frontal theta NFT. The data from one person was excluded due to that person closing the eyes midway during the alpha training session. EEG was recorded from the Fz electrode with the left and right mastoid used as reference and ground. The impedance was kept below 10 kΩ. EEG was acquired at 256 Hz sampling rate and stored for off-line analyses. The software implemented a moving average threshold in which the threshold was set equal to the average alpha power recorded in the preceding minute. A recent theoretical analysis has shown that an adaptive-threshold procedure speeds up learning compared to a fixed-threshold procedure (Davelaar, 2017). In addition, the software produced an additional channel based on a proprietary algorithm that indicated for every timestep whether the signal in the active channel contained an artifact or not, such as maximum of minimum voltages (reflecting loose connections), eye-blinks, and muscle activity.

During training, participants would see a screen with a graph of their EEG timeseries scrolling from right to left, a graph with a yellow bar the height of which represented the alpha power level with a red horizontal line indicating the threshold, and a counter that increases by one for every 250 ms that the alpha level was above the threshold. Every time the counter increased by one, the participant would also hear the sound of a bell.

The experimenter was in the room with the participant throughout the training session. The participant was told that the session was to familiarize them with the neurofeedback setup and that they would be interviewed afterward about their experiences about undergoing neurofeedback. Importantly, the experimenter did not provide any explicit instruction to the participant regarding strategies; rather participants were told to increase the number of counts and bell rings by any mental means they could.

After the training session, the participant completed an explicitation interview (Petitmengin, 2006), which is a systematic approach that enables participants to become aware of a specific experience and to provide a detailed and comprehensive description of it. The interviewer’s task is to work with participants encouraging them to examine and reflect upon the previously unexamined and pre-reflective experience. In this context, the structure of the training session is used to help the participant return to the original experience (a re-enacting or re-evoking process), building up a moment-by-moment picture of how it unfolded. Interviews were carried out by a researcher (JB) who received ongoing training to use the explicitation interview by an experienced phenomenological researcher (VE).

Specifically, each interview opened with the following statement:

In the time we are together, our purpose is to gather a description of what you did during the protocol and my job is to help you do this. I am often going to repeat what you say to me so that you can check that I have understood you correctly and whether anything has been left out. Please feel free to interrupt me at any point. Now, tell me your experience of doing the protocol, whatever it happened to be. As far as is possible, please describe your experience and not what you think your experience was and try not to go beyond what you can report accurately. So, let’s begin, just put yourself back into the situation and tell me exactly what you did.

Throughout, the researcher worked with the participant to clarify and deepen the description and maintain focus. For example, saying, “So, if I understand you correctly, you begin by doing X, is that right?” This can be followed with something like, “And when you do X, how do you do it?” If a participant introduces an image or a feeling, the interviewer can ask what the image is like and work with the participant to find the right word that describes it best, such as is it “clear,” “fuzzy,” “near,” “far” or the location of the feeling, whether it feels “light” or “heavy” and so on.

### Data Analysis and Strategy

Given our research question to compare the subjective experiences between learners and non-learners, we first use the EEG data to group participants according to learning success and then compare the verbal statements of experiences between groups. Our analyses will only provide information about group differences in subjective experiences and not about doing NFT itself. A separate project that uses a big qualitative approach (rather than a “small q” one) will address this latter question.

To determine learning success, the EEG data from each individual were analyzed off-line by band-pass filtering (between 1 and 45 Hz, using a 3-order Butterworth filter) and subjecting a 1-s moving Hamming window (jumping every 0.25 sec) to Fast Fourier transformation. Windows containing more than 2% signal failure were discarded from the analysis, which excluded all gross artifacts. The absolute mean alpha (8–12 Hz) spectral power was extracted from all included windows for each individual. To classify learning success, a cumulative alpha function was generated for each individual. A non-learner was defined as one for whom the alpha power level remained constant throughout a NFT session, resulting in a linear cumulative function. If, however, the alpha power level increased due to successful NFT, the cumulative function would be convex (i.e., becoming larger than linear). We fitted linear and quadratic polynomials to the cumulative functions by minimizing the sum of squared error and compared the goodness of fit, R^2^, adjusted for the number of parameters:

$$R_{\text{adjusted}}^{2}=1-\frac{\left(1-R^{2})*(N-1\right)}{N-p-1}$$

where *N* is the number of epochs per individual and p is the number of parameters for the linear (*p* = 2) and quadratic (*p* = 3) functions. To prevent false positives due to equal adjusted *R*^2^ given the numerical resolution, a difference in adjusted *R*^2^ of at least 0.1% (i.e., the difference between 0.997 and 0.998) was needed to indicate superior fit by the quadratic function.

To provide a tutorial-like approach for the analyses of the interviews, the detailed steps involved in the qualitative analyses are presented prior to their results.

## Results

### EEG Analysis: Determining Learning Success

**FIGURE 1:**
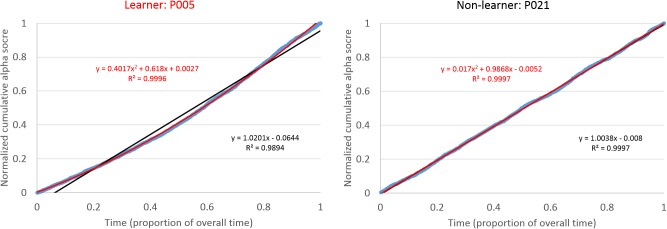
Examples of timeseries of the relative alpha power for the best learner (left) and a non-learner (right) compared to the best-fitting linear function.

To classify participants into learners and non-learners we fitted linear and quadratic polynomials to the individual cumulative alpha functions. This resulted in four participants being identified as learners, as their cumulative function was best fit by a quadratic polynomial. To visualize the fits, we show an example of a learner and a non-learner in Figure 1, demonstrating the deviation between the empirical data and the best-fitting linear function. Individual fits are presented in the Supplementary Figure S1. One possibility is that the fitting method produces stronger learning effects for those participants that start off with low levels of alpha power. However, this was not the case, as the correlation between the quadratic slope and the average alpha power of the first decile was not significant (*r* = −0.31, *p* = 0.24).

**FIGURE 2:**
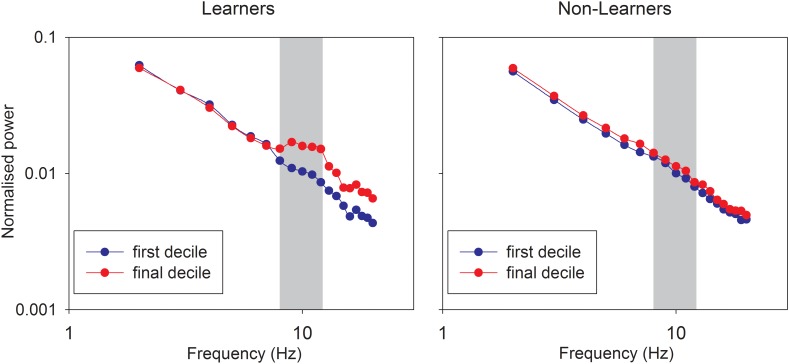
Log-log plots of the normalized frequency spectra for learners (left) and non-learners (right) for the first and final decile of the timeseries. The shaded area represents the frequency band (8–12 Hz) that was targeted during the (eyes-open) training session.

Figure 2 shows the average normalized spectrum plots for the first and last decile of the two groups, demonstrating that the learners did indeed learn to enhance the relative power in the alpha band. Individual first-last spectra and time-frequency plots are provided in the Supplementary Figures S2, S3.

### Interviews

The interviews were transcribed and statements were extracted that were related to mental states during NFT. These statements were then interpreted by creating a classification system that resonates with a cognitive-theoretical framework. This choice allows a closer link between the quantitative methods/philosophy used in cognitive neuroscience and the methodology used in qualitative research. The resulting classification makes a distinction between what trainees were doing (actions, aka strategies), what they sensed (percepts), and what executive functions they performed. The interviews indicated that each of these classes could be further divided into specific subclasses. Table 1 shows the classification together with examples of statements that lead to this class. Note that not all participants provided information for all subclasses.

**Table 1:** Classification system developed based on statements from the transcribed interviews with examples.

Classification	Examples
(1) Actions (16)	
Cognitive (16)	I try to focus on the here and now (P001)
Bodily (14)	I was mainly focused on my breathing (P009)
(2) Percepts (16)	
Emotion/mood/feeling (13)	I was excited (P021)
	I was getting really annoyed (P017)
Embodiment (9)	Something inside my stomach (P011)
Other senses (10)	*Auditory*: I was aware of the sounds of the beeps (P004)
	*Visual*: I could see myself as if somebody’s looking at me (P009)
Cognitive (8)	I ran out of things to think (P010)
	Sometimes things just pop in and it just gets messy again (P006)
(3) Executive (14)	
Change in actions (3)	I switched to doing math in my head (P002)
Change in percepts (1)	I kind of forgot I was in the chair (P013)
Evaluative (12)	I wouldn’t say not caring, but not minding whether the bar was moved actually seems to be more effective (P015)
Goal-directedness (2)	I was conditioning myself to push on (P005)

*The number of participants (out of 16) that provided statements for that (subclass) is given in brackets. The code after each example statement refers to the participant that provided that particular statement.*

### NFT Success and Subjective Experience: A quantitative Analysis

The individual statements in the classification system were integrated into a n x m matrix, with *n* = 16 participants and *m* = 202 unique statements. The statements were labeled using the developed classification system and specific word choices provided by participants. This produced 87 unique labels. We then scored the presence/absence of a label, such that each participant was only scored once per one unique label. For example, “I tried to think of what I’d be doing after the session” and “I thought of what I’d be doing on my holiday” are both labeled as cognition-planning. This filtering produced 169 unique label counts across the participants. We then clustered the labels into larger topics, ignoring specifics. For example, the labels “feeling-happy” and “feeling-anxious” were clustered under the topic “emotion”. This produced a total of 15 topics. Finally, we maintained only those label-clusters for which there were at least 4 instances, which on average means that each person could have contributed to that topic, as topics that are mentioned by only one person may not be generalizable. This was the case for 8 topics that covered a total of 159 topic-instances. Within this reduced dataset, the number of topic-instances per participant ranged from 4 to 18 with an average of 10.2 and 9.3 for non-learners and learners, respectively.

**FIGURE 3:**
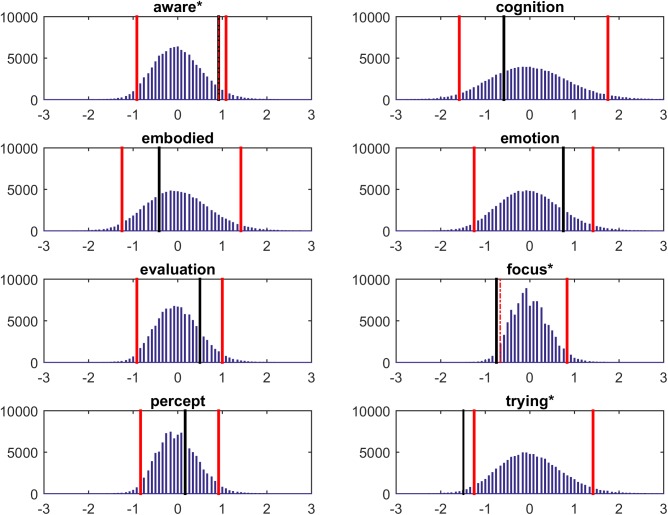
Bootstrapped frequency null-distributions of the differences in counts of the topics of experience shown above each panel. The observed data pattern was bootstrapped 100,000 times. The solid black line indicates the value of the data. Positive values reflect that learners were more likely to report on the experience than non-learners and vice-versa. The solid red lines reflect the criterion levels for two-tailed significance levels of 0.05. The dashed red lines for the *aware* and *focus* topics reflect the criterion level for one-tailed significance. The three starred topics are candidate topics that distinguish between learners and non-learners. See text for further discussion.

**FIGURE 4:**
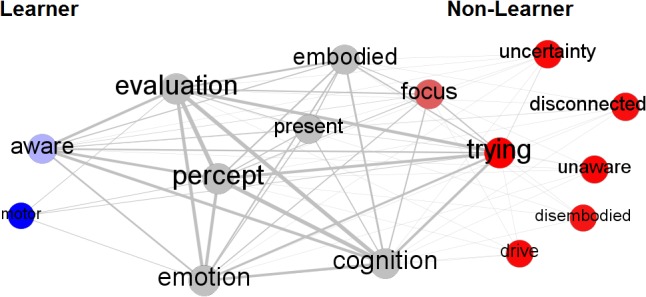
Network visualization of the topics reported by learners and non-learners. The size of each node is proportional to the number topic-instances. The thickness of the internode connections reflect the frequency with which the two topics are present in verbal reports. Red nodes are topics that are reported more often by non-learners compared to learners and vice-versa for the blue nodes.

Due to the low number of learners and the need to compare the frequencies of statements across groups, we used a bootstrap procedure. For this, we simultaneously randomized the topic-instances and the participants while keeping the same distribution of instances/participant and instances/topic (84 × 16 matrix). We then recalculated the between-group difference in average number of instances per topic. This was repeated 100,000 times to create a null-distribution for each topic. Figure 3 shows the bootstrapped null-distributions for each topic. The observed between-group difference was then compared to the null-distribution by calculating a *z*-score (under the bootstrapped null) and the associated *p*-value. We report the significant two-sided *p*-values, as we did not have *a priori* hypotheses. However, given the exploratory nature of this study, significant one-sided tests are presented as well for reference. Table 2 shows the eight topics for which at least four instances exist in the dataset. The remaining seven topics are shown in Figure 4 and were: “disconnected,” “disembodied,” “drive,” “motor,” “present,” “unaware,” “uncertainty.” Figure 4 shows a network visualization of all topics with red and blue topics being mentioned more often by non-learners than learners, and vice-versa, respectively. The edges connecting topics reflect their co-occurrences within verbal reports. Thus, when a person reports “trying to relax” and “feeling excited”, an edge is added between *trying* and *emotion*.

**Table 2:** The eight topics (with at least four instances) with the number of topic instances per group and the relative group difference together with the associated z-scores from the bootstrapped null-distributions.

Topic	*N*_resp, learners_	*N*_resp, non-learners_	*S*_difference_	*z*-score	
Awareness	6	7	0.9167	1.7299	*p*_one-tailed_ = 0.0418
Cognition	8	31	−0.5833	−0.6873	
Embodiment	2	11	−0.4167	−0.614	
Emotion	7	12	0.75	1.0989	
Evaluation	6	12	0.5	1.0147	
Focus	0	9	−0.75	−1.8188	*p*_one-tailed_ = 0.0345
Perception	6	16	0.1667	0.3649	
Trying	2	24	−1.5	−2.1853	*p*_two-tailed_ = 0.0144

*The p-values of the significant z-scores are shown. N_resp, learners_ and N_resp, non-learners_ refer to the total number of instances for the group of learners (n = 4) and non-learners (n = 12).*

### NFT Success and Subjective Experience: A (Very Small) Qualitative Analysis

To complement the quantitative analysis, we conducted a (very) small qualitative analysis. Specifically, we created structural descriptions based on the statements for each group that captures most of the participants in each group. Thus, the main aim here was to produce descriptions that capture those experiences that do not vary across participants. Although typically a general structural description is produced by first creating one for each participant and then integrating them, due to the large individual differences in focus and tangential detail combined with the relatively short interview session, a different procedure was used.

Specifically, for each person a summary from the transcribed interview was created, which may take the form of a few sentences or statements. Next, the 202 statements were ordered by group and topic frequency and then condensed using the individual sentences from the interviews. This stage allowed for identification and exclusion of idiosyncratic statements. In addition, the ordering of the sentences in the descriptions follows the topic frequency and thus accentuates the relative importance of the content within the respective group. For example, the first sentence, “I tried various things” captures the majority of non-learners. Finally, to emphasize these group differences, a concluding sentence was added (i.e., “Overall,...”) which points to the key salient qualities of the interviews. The phrases used in the descriptions are those used by the participants.

A structural description for non-learners (*N* = 12) reads:

I tried various things. I tried to empty my mind and not really think of anything at all. Then, I would remember events or people to block out thoughts coming into my mind and distract me. I also tried to relax by controlling my breathing, taking slow deep breaths. I concentrated on the bar and will [sic] it to move. I picked a place on the screen to focus on and it went blurry; my eyes were open but I could not see anything. I could still hear the bell. I was anxious, but also relaxed and excited. I could feel my breathing and focused on it. I was aware of my thoughts all the way through. Overall, I focused mostly on trying to get the bar to move by finding a pattern for the behavior of the yellow bar reaching the red line.

A structural description for learners (*N* = 4) reads:

I was trying not to think of anything by staring at something on the screen until I got fuzzy-eyed. I also tried to concentrate on something in the room, but in a relaxed way. In my mind, I switched from counting sheep to naming colors to doing math. I felt really calm and relaxed, but also happy and excited. I was aware of the beeps all the time and of your presence in the room. I tried to put tension in my body and raise my heart rate. Overall, I was aware of my surroundings and made sure I was in the moment as much as possible.

As can be observed, learners and non-learners report and emphasize different aspects of their experiences. These descriptions ignore, as much as possible, the idiosyncratic variation and redundancy to capture the invariant structures. Importantly, these integrative narratives combine both quantitative and qualitative information at the within- and between-group level. By and large it converges with the quantitative analysis of this data, which will be discussed next.

## Discussion

Neurofeedback is a powerful tool to help individuals control their brain profiles to optimize their mental functioning and well-being. However, not everyone manages to reach their potential level of control. We assumed that this is partly a consequence of different learning approaches to neurofeedback, some of which are congruent and some of which are incongruent with the learning goal. If neurofeedback practitioners, trainers, and coaches know what type of mindset or attitude supports the desired neurofeedback goal, NFT success could be facilitated in not only late-learners, but also those who would otherwise be classified as non-learners.

In this study, we investigated the mental mindset of individuals who experienced a single 5-min NFT session to increase their alpha frequency over the frontal cortex, which has been shown to be related to improved cognitive control (Ros et al., 2013; Berger and Davelaar, 2018). The short session allowed a large number of individuals to be classified as non-learners to which the smaller number of learners could be compared. Interviews conducted immediately after the training session revealed that learners and non-learners do indeed entertain different mindsets as obtained using both quantitative and qualitative analyses. In our sample, those who managed to increase their alpha power were mainly describing being aware of something. Those who failed to enhance their alpha band frequency were mostly investing mental effort in the acts of trying or exerting deliberate attentional focus. This includes mental actions, such as “trying to relax,” “trying to empty the mind,” and “focus on breathing”. We can tentatively redescribe the experiences as falling on a trying-sensing continuum, with learning success increasing when going from a mindset of trying or exerting cognitive effort to sensing. At this stage, we are not proposing a mechanism; rather we speculate what could be interpreted from the findings. So, it is critical to note that in all the papers that address subjective experiences (see introduction), the emphasis is on doing something: a strategy. Our results show that sensing is as important as and arguably more important than trying. This echoes the results of Garrison et al. (2013) using fMRI where both actions and awareness were basic factors related to PCC activation.

Although increased alpha has been linked to enhanced attentional focus and cognitive performance (e.g., Klimesch, 1999; Jensen et al., 2002; Cooper et al., 2003; Sauseng et al., 2005; Klimesch et al., 2007; Berger and Davelaar, 2018), here we found that *learning to increase* alpha is associated with an absence (or restriction) of an evaluative process that operates on the products of sensory and cognitive processes. Thus, a cortical area could be in a heightened state of attention to augment sensory or cognitive information, in the absence of emotive-evaluative processing.

Despite many critical differences between our study and that by Edge and Lancaster (2004), some useful comparisons can be made. In the Edge and Lancaster study, one participant described being in a relaxed state, but still able to exert enough control to prevent falling asleep. In our study, one of the responders said that she was tired or drowsy. The theme of “experiencing dreaming, images, or fantasy” discerned by Edge and Lancaster (2004) was not obtained in our study. A likely possibility is that this particular theme is either associated with the theta component of the alpha/theta protocol or the electrode placement at the back of the head or both.

### Strength and Limitations

Our study extends the database by contrasting learners with non-learners in a single study and focus entirely on the experience of a short neurofeedback session. The richness of the results is testament to the utility of the (neuro)phenomenological approach and gives empirical support to viewing “neurofeedback as a new bridge between neuroscience and phenomenology,” as put forward by Bagdasaryan and Le Van Quyen (2013). However, this study is still preliminary with specific strengths and limitations.

In contrast to previous studies which use questionnaires to obtain subjective information, a strength of the current study is the use of an explicitation interview to get close to the lived experience. In addition, care was taken not to bias the interviews through the use of constant clarification, summarizing and reflecting back on the part of the interviewer. Procedurally, participants were told about the impending interview unlike in Edge and Lancaster, resulting in more qualitative material to work with. The current work allowed the initial development of a classification system which has the potential for further research to map the space of subjective experience and neurofeedback. This method can be applied to any NFB protocol, whether the results will be the same or not is an open and interesting question.

A possible limitation of the present analysis is that it is restricted to a single short NFT session. However, from a clinical perspective a short session allows the clinician to assess whether the client has the protocol-appropriate stance (for alpha: trying-sensing). The clinician could then instruct the client to adopt the appropriate approach instead of wasting an entire training session. It is of considerable interest to conduct the analyses across multiple sessions to track the change in subjective experience. Gruzelier et al. (2010) found associations between subjective feeling of change and EEG recordings at the level of sessions. Thus, it is not impossible that subjective experiences evolve at multiple timescales. Kober et al. (2013) noted that learners were those who went from reporting any strategy after the first session to reporting no strategy after the tenth session. However, no detailed interview was conducted to allow a qualitative analysis.

In the fMRI study by Garrison et al. (2013), highly skilled meditators were tested and were instructed in the self-reporting. No interviewer was present for deepening the verbal reports. The instructions explicitly focused the participants to find associations between a summary graph, showing the brain activation from beginning to end compared to baseline, and their recollected experience. In addition, the PCC is a brain area that is known to be involved in self-related processing (Mason et al., 2007; Guterstam et al., 2015), which may make it easier to discover neuro-experiential profiles. As we did not have individuals who were skilled in self-reflection, we utilized the explicitation interview. This led to the bottom-up creation of the aforementioned classification system, as no top-down series of questions (even open-ended ones) were imposed. Of interest, the verbal reports obtained by Garrison et al. (2013) were qualitatively analyzed in an analogous manner as in our study. That is, the brain data was used to provide objective classification of the phrases in PCC activation and deactivation, followed by further merging into higher-order classes. The analyses demonstrate the utility of detailed verbal reports to extract subjective experiences and relate them to brain patterns.

We took a quantitative approach to qualitative data. Here, this is appropriate as the research question of exploring *differences* in subjective experience is congruent with the approach of “bean counting” in which the number of certain phrases is counted for each group separately and compared (see e.g., Nowlis and Kamiya, 1970). We went a step further by using a bootstrap procedure to perform parametric statistics to get some indication of replicability for further work. The marginal significance of “awareness” and “focus” make these experiences ideal candidates for further qualitative study.

Although in Garrison et al. (2013) study and in our current one the verbal reports and neural events are separated by minutes, Petitmengin and Lachaux (2013) discuss the possibility of separations of seconds. They describe the use of intra-cerebral recordings with patients where feedback is presented about the neural activity over the preceding 10 s. Using the explicitation method a temporally closer proximity between subjective experience and neural patterns can be established, which in turn can facilitate neurofeedback learning. More studies employing both first- and third-person methodologies are needed to compare and integrate results across imaging modalities, brain regions, and self-reporting skills.

### Further Research

This research relies heavily on participants’ ability to reflect on recent past experiences without interpreting their experiences and on interviewers’ ability to direct the participant to provide an as complete picture of the experiences as possible. Due to the preliminary nature of this project, no concrete guidelines were available that were tailored to the neurofeedback environment. As such, there were some missed opportunities regarding topics mentioned by participants that were not unpacked and topics not mentioned by participants that could have been asked if a classification system existed. In this regard, this study was a success for the creation of both a classification system and an integrated mixed methodology. Follow up studies could benefit from the developments piloted in this paper. One such approach could be to conduct a semi-structured interview based on the classification system in order to extract as much information about the experiences as possible. For neuroscientists who cannot include interviews or qualitative analyses in their studies, phenomenology-derived questionnaires could be constructed that probe many of the relevant topics mentioned by the trainees, but missed in past research due to it not being a cognitive strategy or captured by a particular label on an existing questionnaire.

Given our aim to investigate what differences in mind set exists between alpha learners and non-learners, our tentative recommendation to neurofeedback trainers is to instruct participants to be aware of various things, but not aim to anticipate or predict the feedback. To test this hypothesis, we are currently running a study addressing whether a pure instructional manipulation that guides the person toward reaching the desired subjective experience is successful in enhancing frontal alpha. Such an instruction would be akin to instructions for meditation training. One would assume that those who are more mindful would be able to learn the neurofeedback protocol faster. For example, recent evidence (Cassady et al., 2014) demonstrates that people with experience in mind-body awareness training (MBAT) have a greater affinity with brain-computer interfaces than those with no MBAT experience.

Further work could address the replicability of the association between learning success of frontal alpha and subjective experience, especially when using multiple training blocks in a single session or extending the protocol to involve multiple sessions with interviews at the different stages of the training program. Follow up work could also apply the methodology and analyses to other neurofeedback protocols and adopt a more principled qualitative approach which is more bottom-up driven and that is more aligned with the philosophy of qualitative research.

## Conclusion

We take the view that EEG states have an associated subjective state, which can be used to learn to voluntarily enhance frontal alpha power. To facilitate research in this area, we developed a classification system that can guide semi-structured interviews and data analyses. We also demonstrated how both quantitative and qualitative methodologies can be integrated to provide insights in the differences between learners and non-learners. Finally, the surprising result that the typical instruction of “trying to relax” may actually be detrimental to NFT success should inspire researchers to expand on the work presented here both at the level of the chosen protocol and the depth of analysis. Inevitably, this would require integrating phenomenological methodologies and analyses into the neurofeedback research paradigm.

## Ethics Statement

The research was approved by the Local Departmental Ethics Committee. All participants provided written consent prior to the study and also additional verbal consent prior to interview for having it recorded.

## Author Contributions

ED and VE designed the study. SA and JB collected the data. ED, VE, SA, and JB analyzed the data. ED drafted the initial paper. VE, SA, and JB helped in revising. All authors approved the current version and were accountable for the accuracy of the results as presented.

## Conflict of Interest Statement

The authors declare that the research was conducted in the absence of any commercial or financial relationships that could be construed as a potential conflict of interest.
