# Supplementary material for: Differential Subjective Experiences in Learners and Non-learners in Frontal Alpha Neurofeedback: Piloting a Mixed-Method Approach
Source: Front Hum Neurosci. 2018 Oct 23;12:402. doi: 10.3389/fnhum.2018.00402 (PMC6206258; doi:10.3389/fnhum.2018.00402)
Supplement: Supplementary file 1 [file Data_Sheet_1.docx]

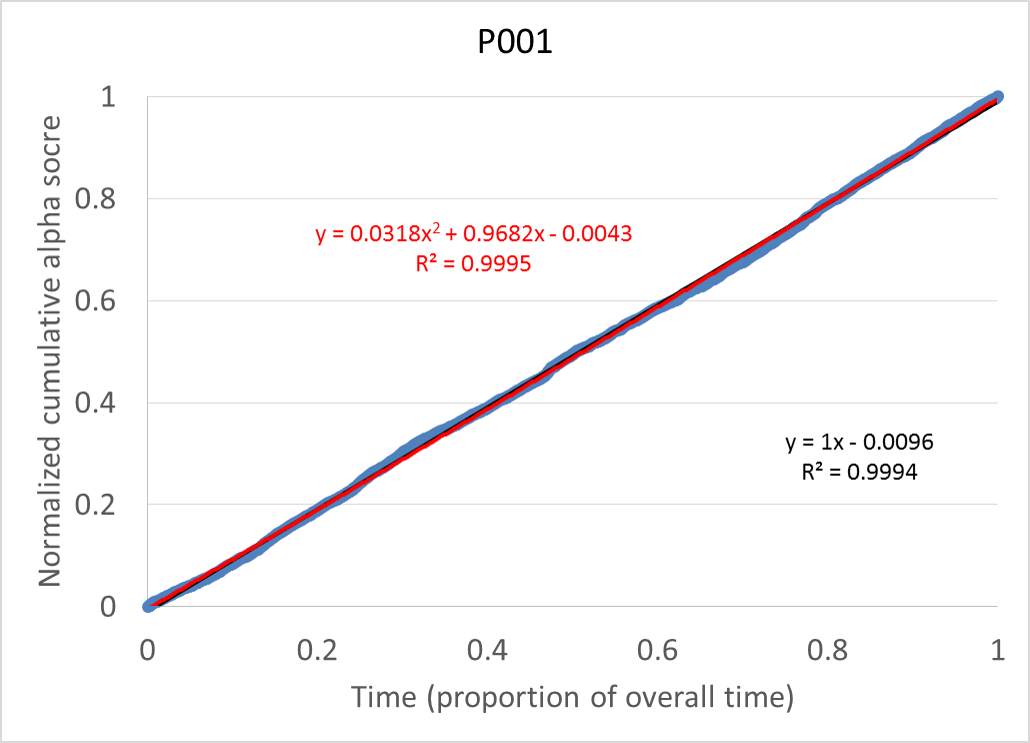


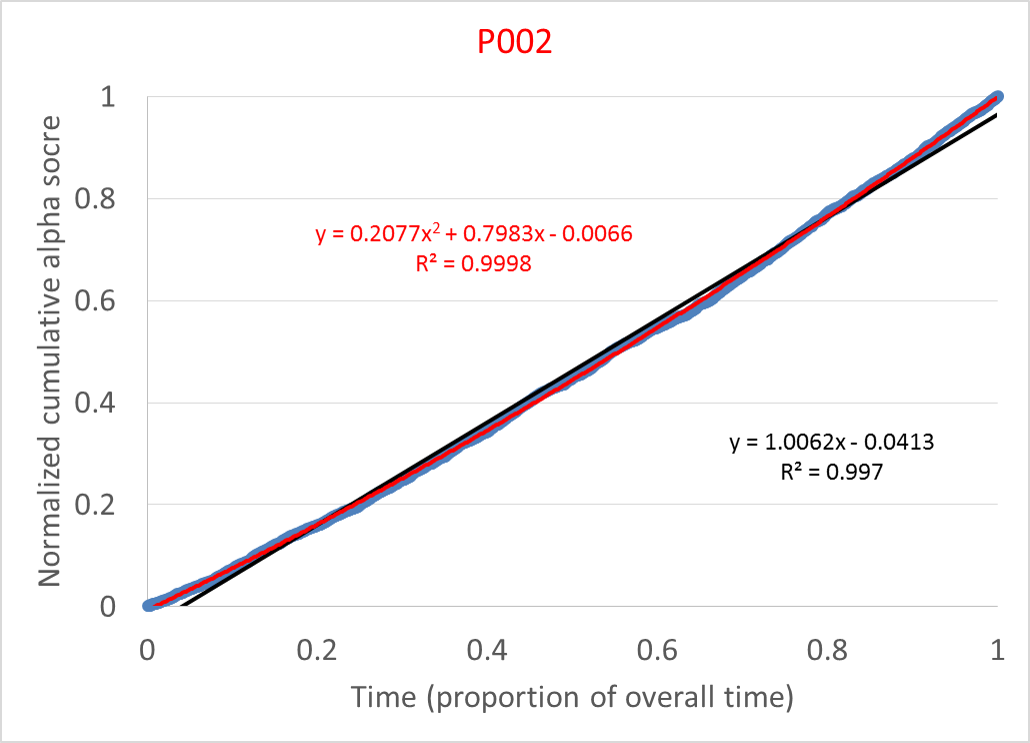


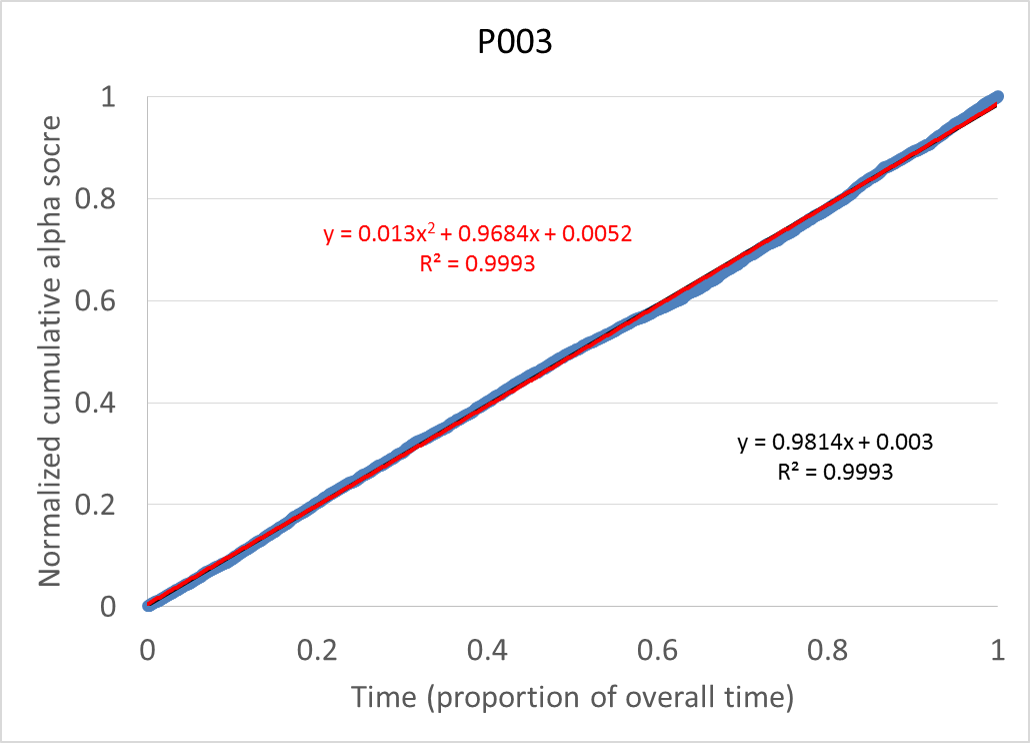

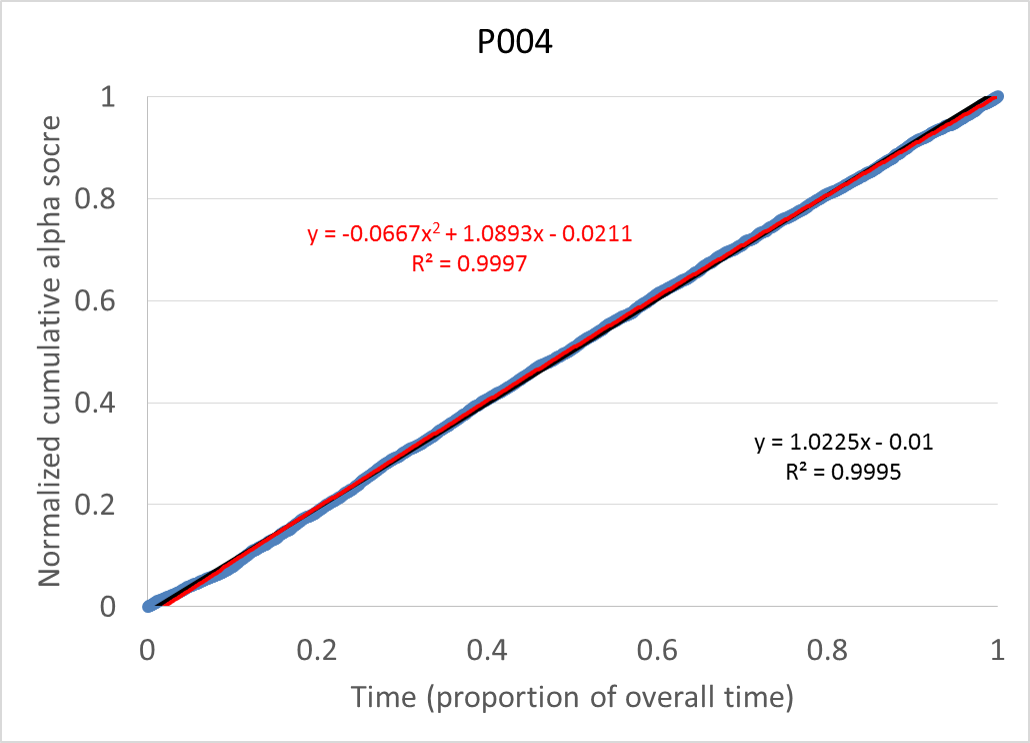


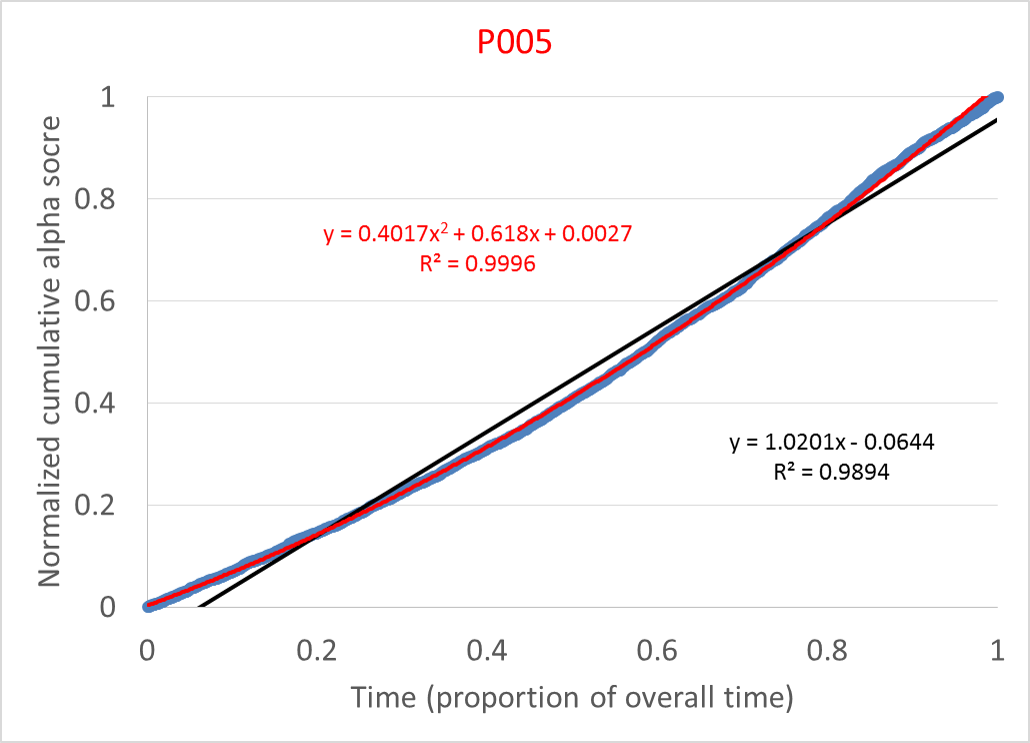

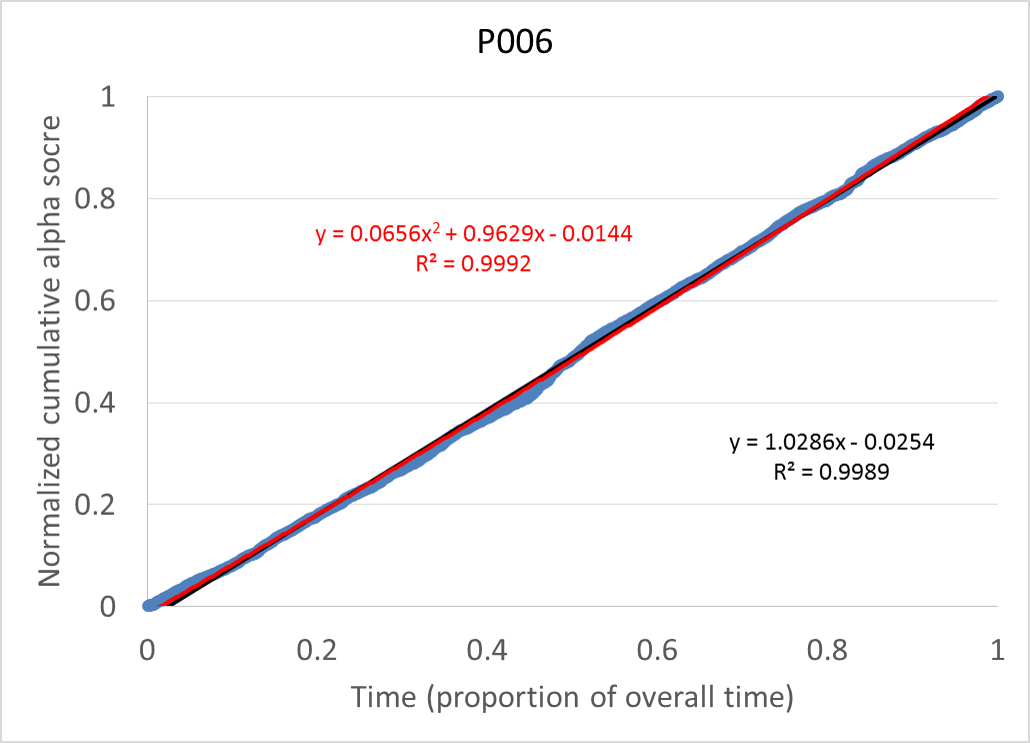


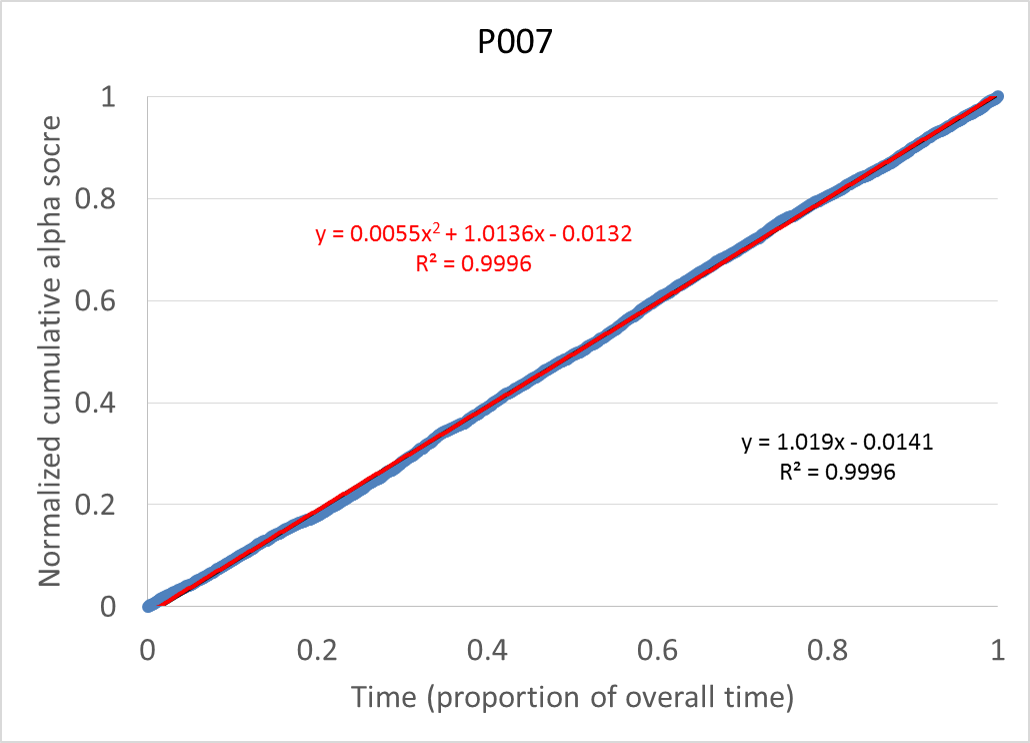

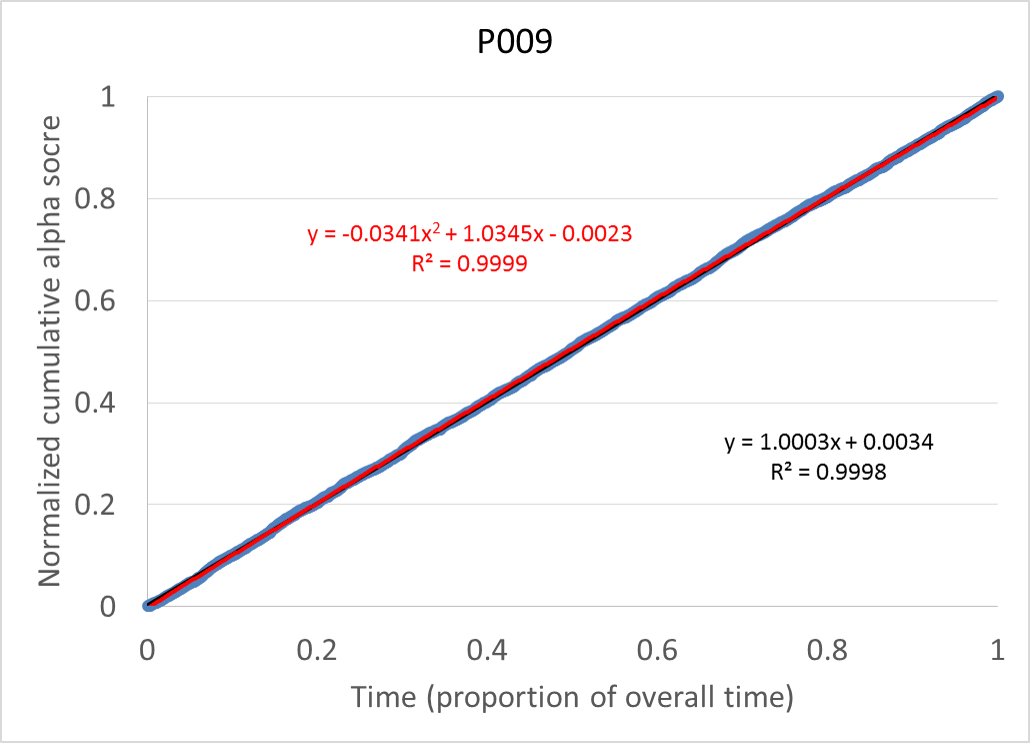


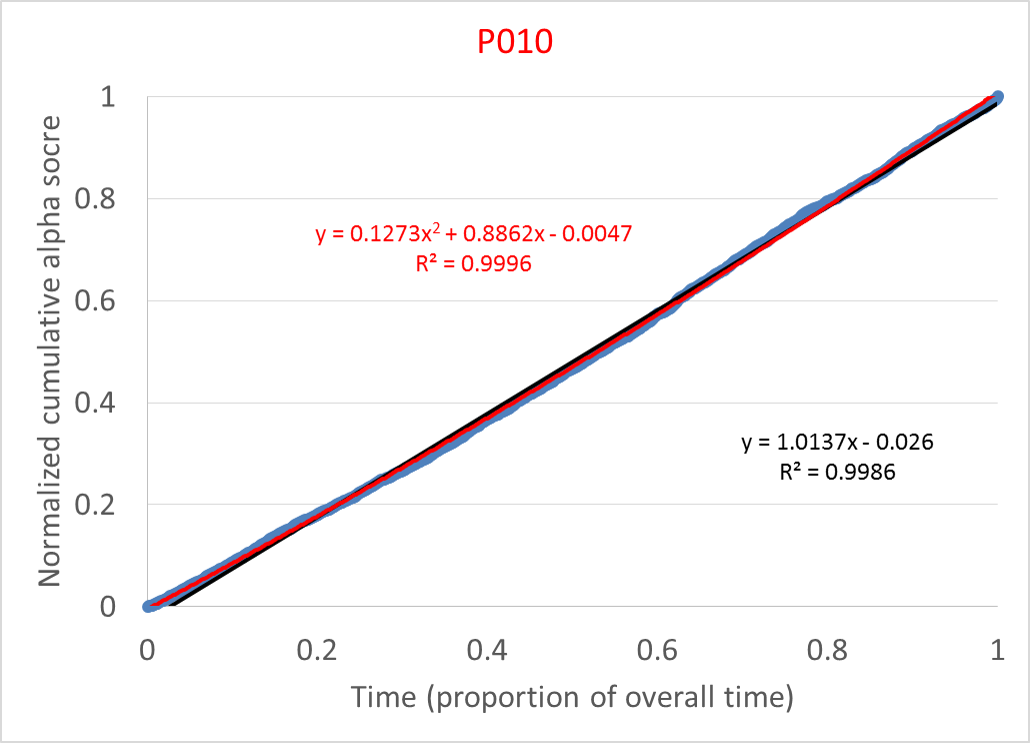

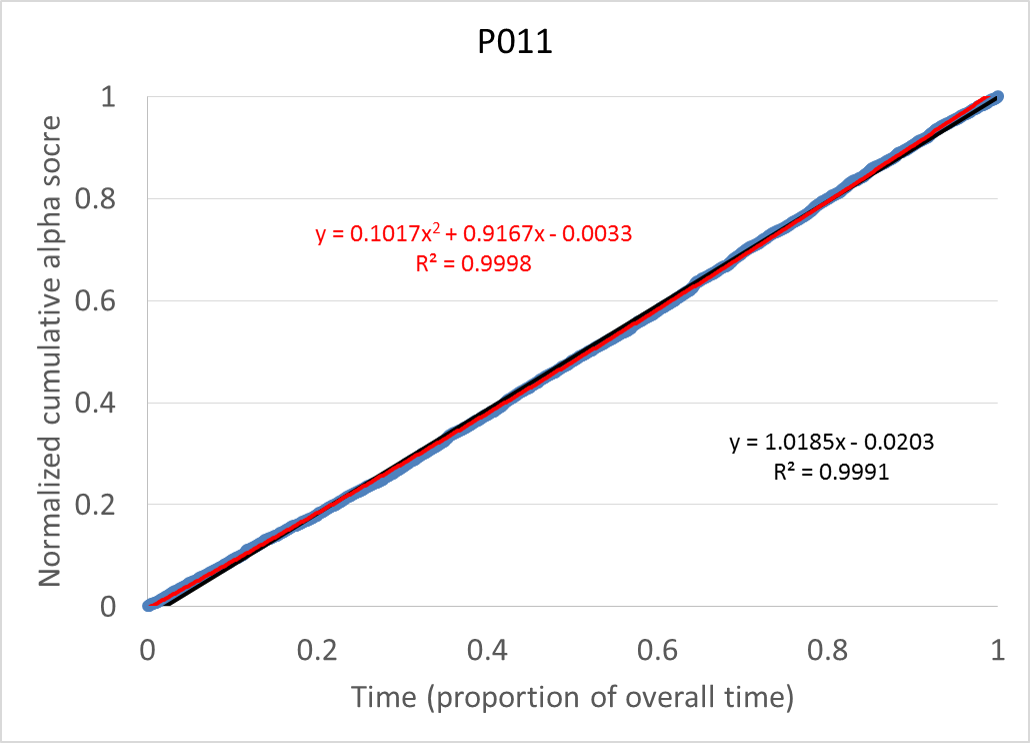


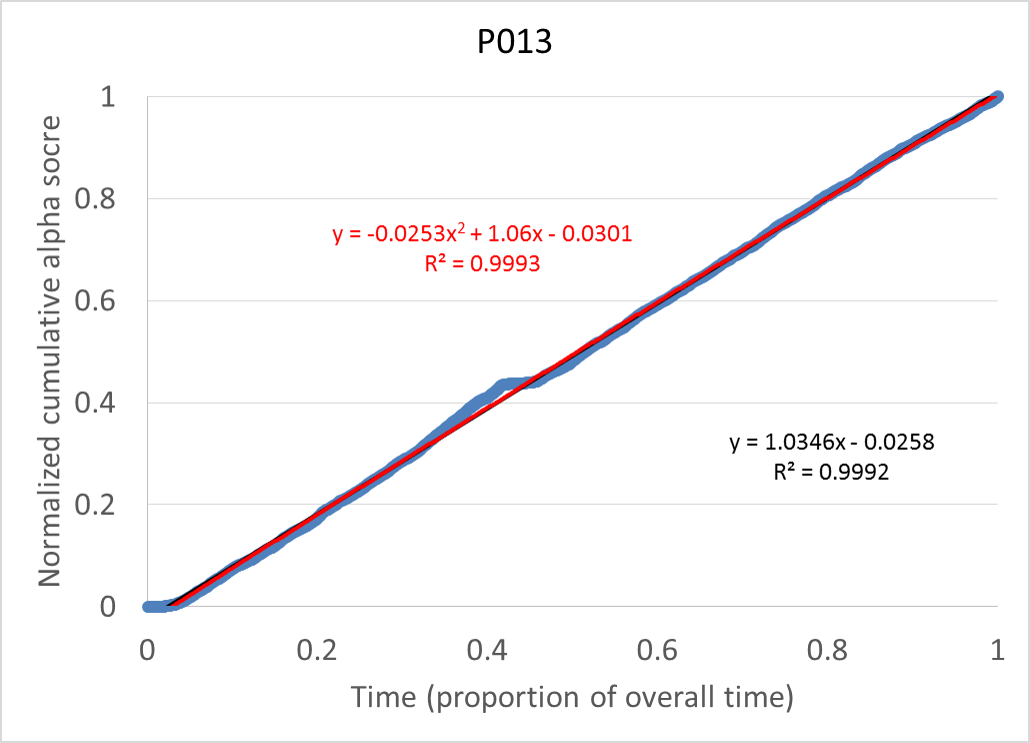

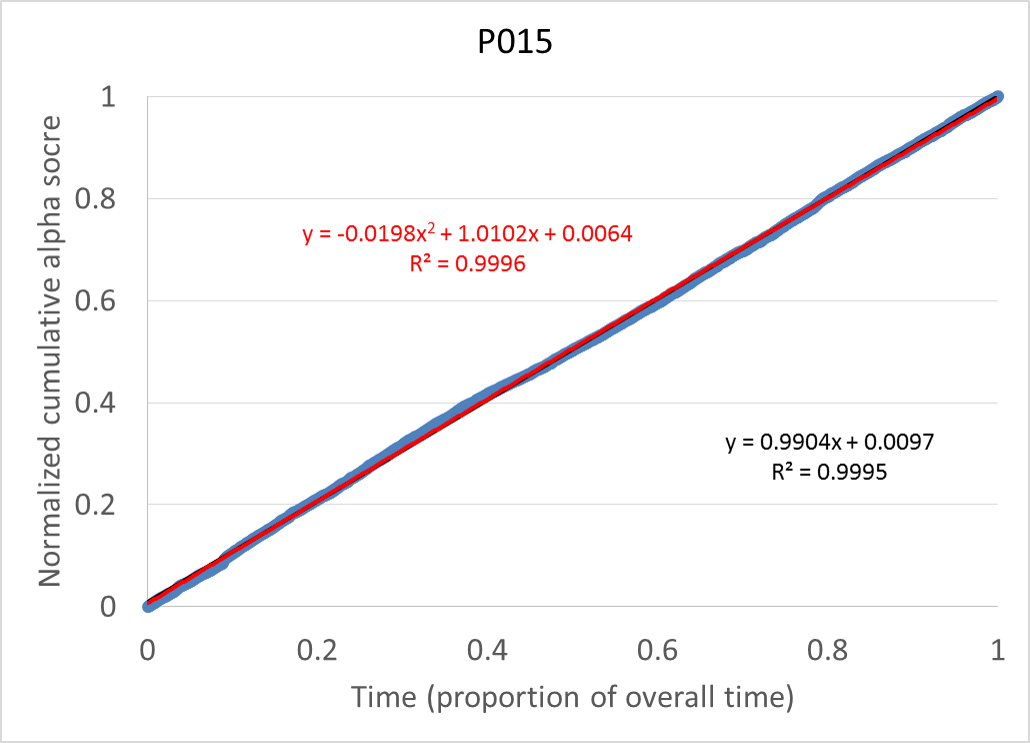


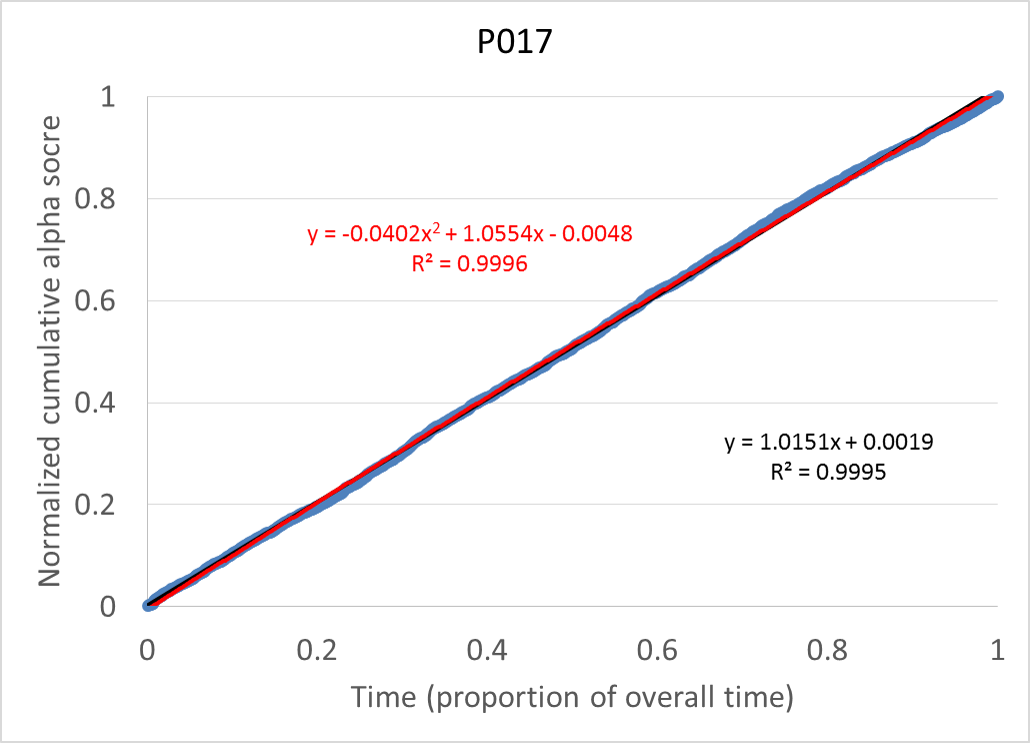

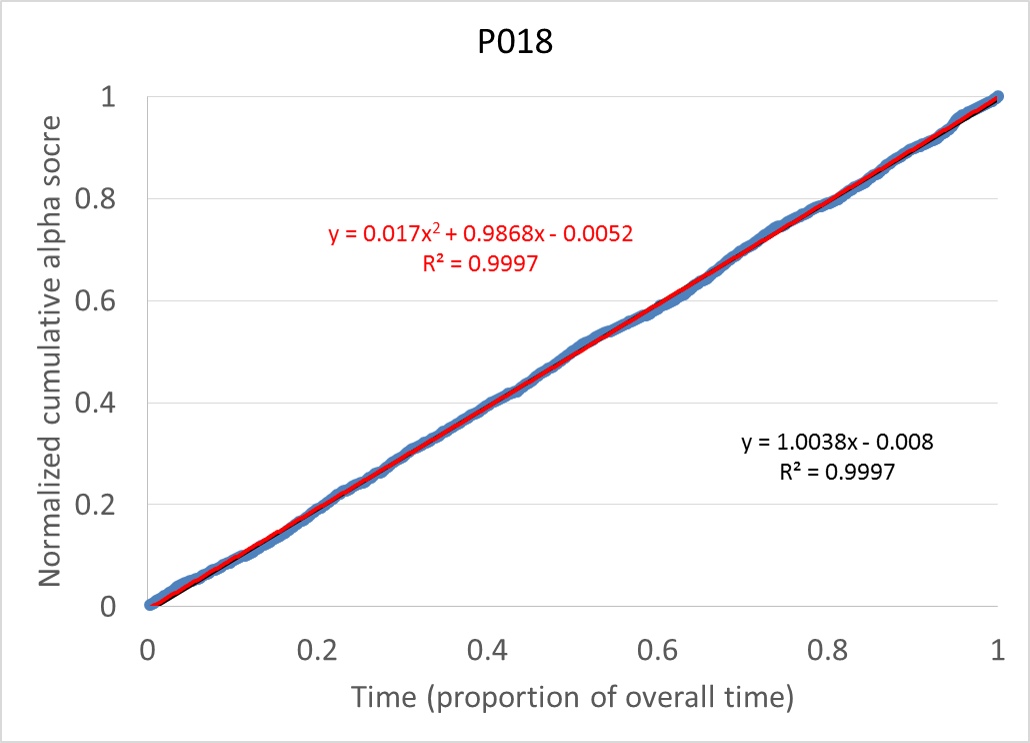


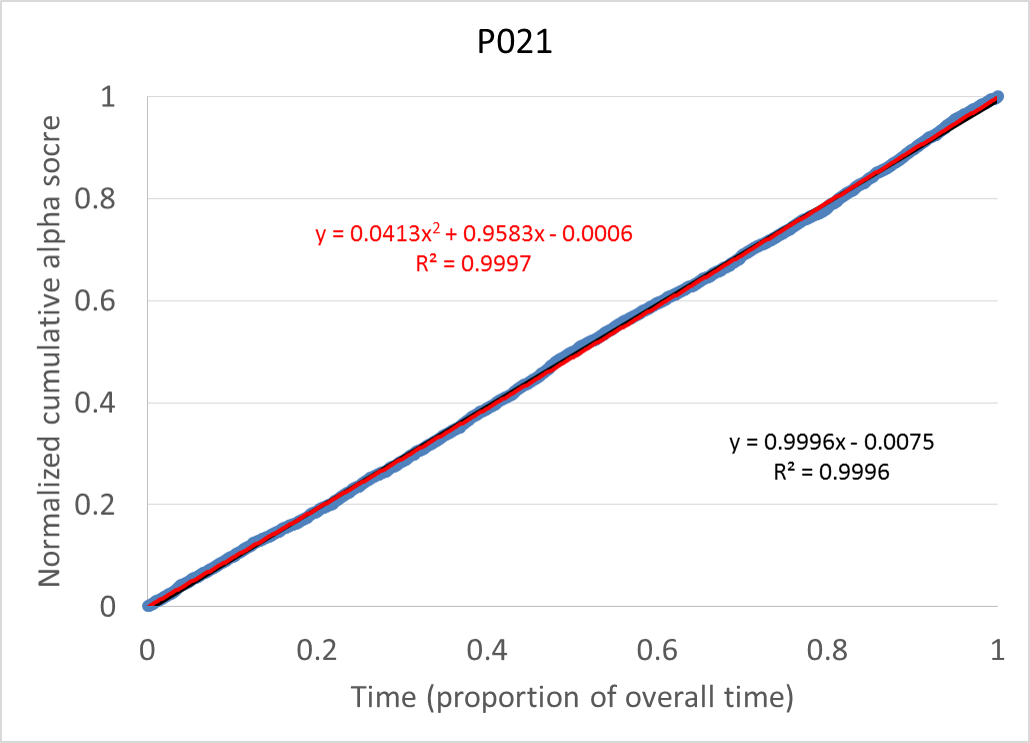

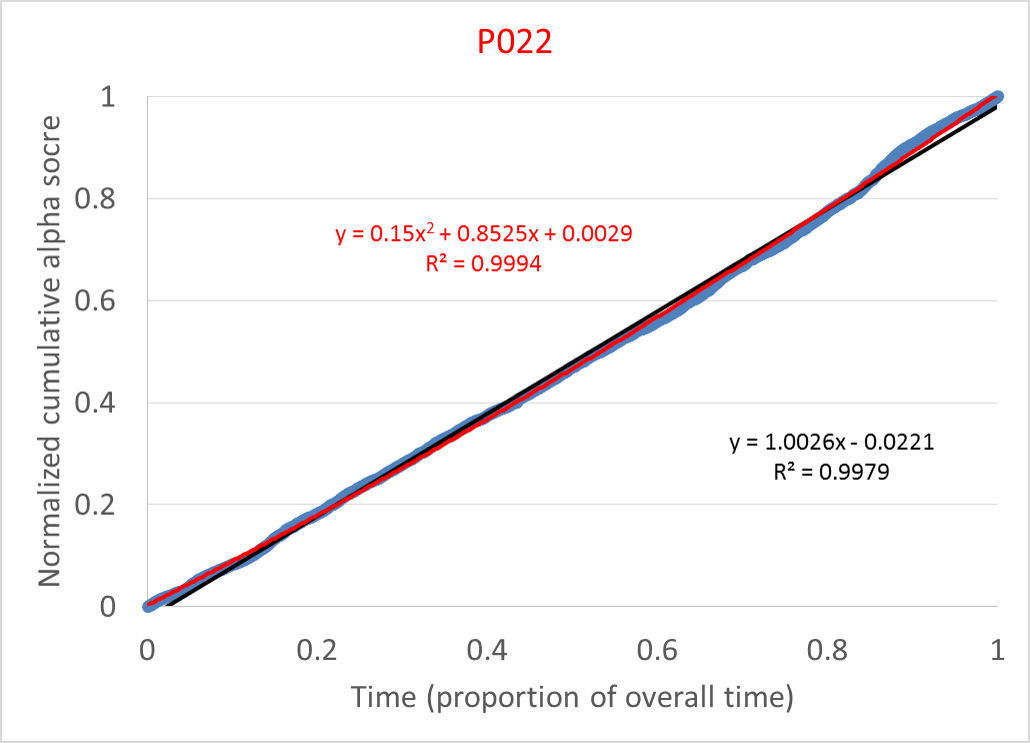
 Figure S1. Timeseries of the relative alpha power compared to the best-fitting linear function for all individuals separately. The plots from learners are indicated with the red participant code. Example data are shown in Figure 1 of the main text.


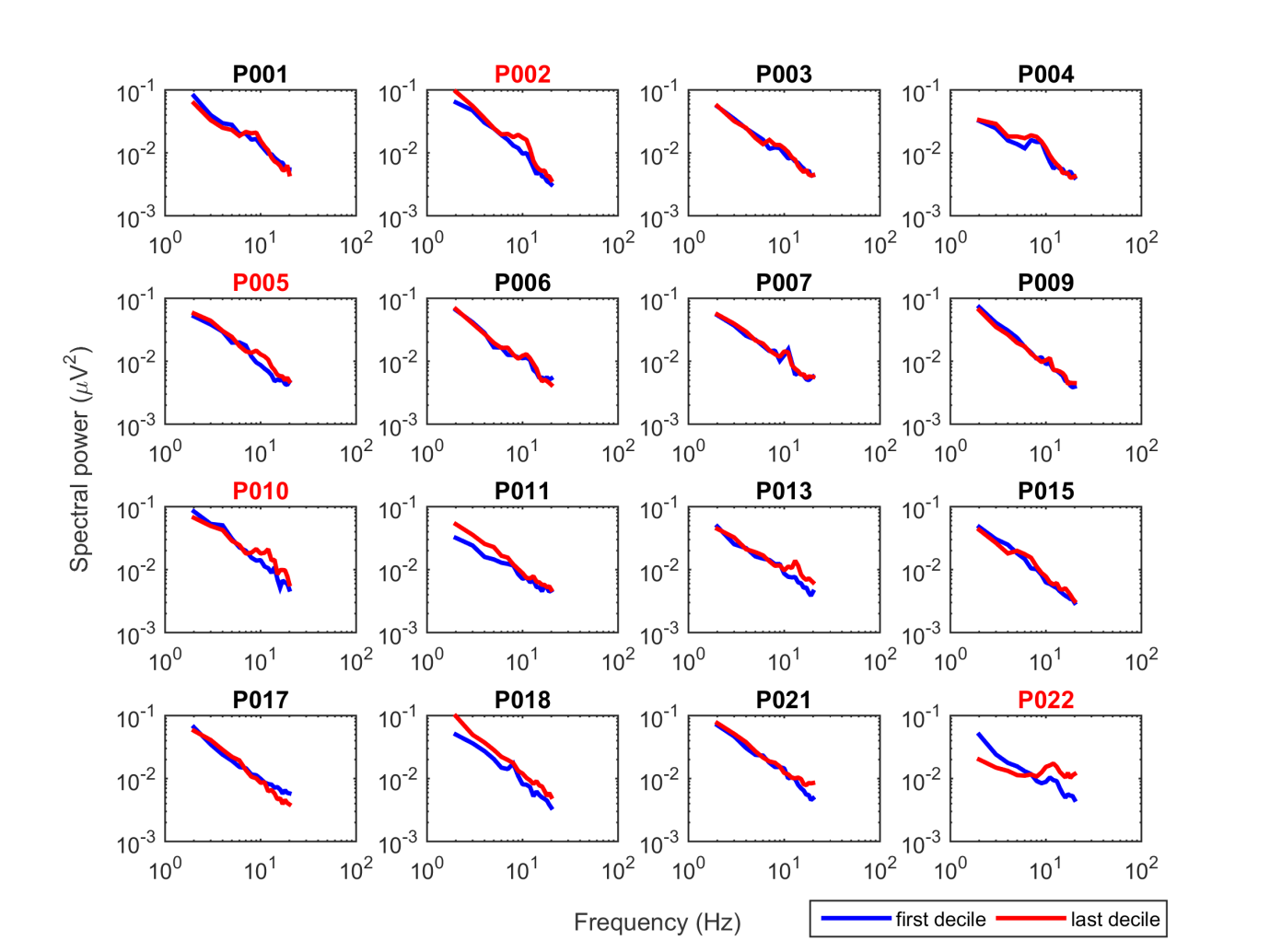


Figure S2. Log-log plots of the normalized frequency spectra for all individuals separately for the first and final decile of the timeseries. The plots from learners are indicated with the red participant code. The grouped data are shown in Figure 2 of the main text.


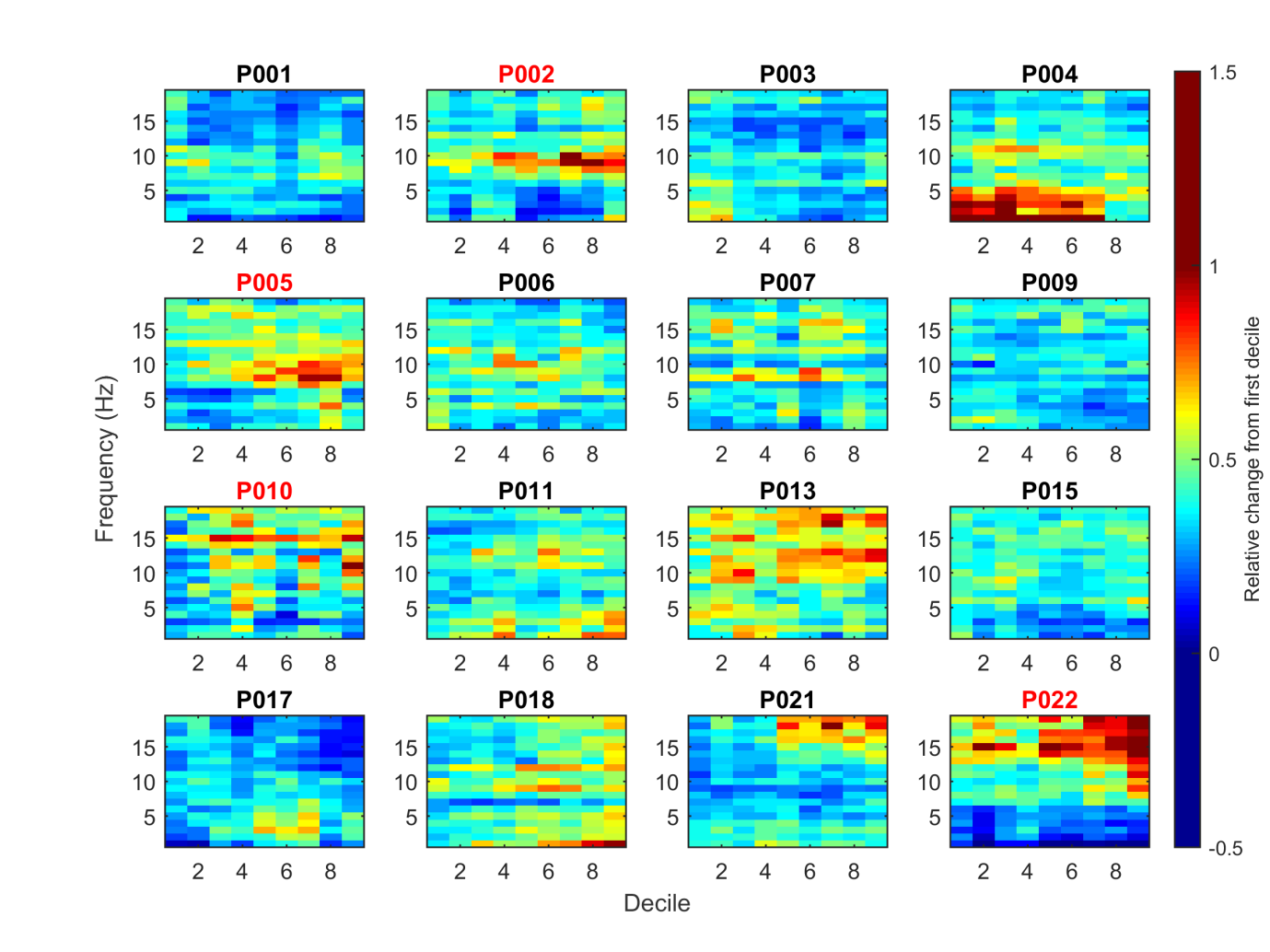


Figure S3. Time-frequency plots for each participant normalized to the first decile. Each epoch represents one decile of the participant’s timeseries. The plots from learners are indicated with the red participant code.
